# The effect of acute complicated appendicitis on liver function test

**DOI:** 10.12669/pjms.37.2.3356

**Published:** 2021

**Authors:** Kulsoom MoulaBux, Sughra Parveen, Mazhar Iqbal, Ayesha Mehboob

**Affiliations:** 1 Dr. Kulsoom MoulaBux, MBBS, FCPS-I. Department of General Surgery, Ward-3, Jinnah Postgraduate Medical Center, Karachi, Pakistan; 2 Dr. Sughra Parveen, MBBS, FCPS, FRCS. Department of General Surgery, Ward-3, Jinnah Postgraduate Medical Center, Karachi, Pakistan; 3 Dr. Mazhar Iqbal, MBBS, FCPS. Department of General Surgery, Ward-3, Jinnah Postgraduate Medical Center, Karachi, Pakistan; 4 Dr. Ayesha Mehboob, MBBS, FCPS-I. Department of General Surgery, Ward-3, Jinnah Postgraduate Medical Center, Karachi, Pakistan

**Keywords:** Hyperbilirubinemia, Gangrenous appendicitis, Suppurative appendicitis

## Abstract

**Objectives:**

To determine the diagnostic role of hyperbilirubinemia in acute appendicitis like suppurative and gangrenous appendicitis.

**Methods:**

This observational study was conducted at Ward-3, Jinnah Postgraduate Medical Center (JPMC), Karachi from 1^st^ June 2019 to 1^st^ June 2020. Males and females above 12 years of age were included. Serum liver function tests and leukocyte counts were carried out. Appendectomies were done, the operative findings and histopathology reports were noted. Hyperbilirubinemia was related with the stages of appendicitis. Results were analyzed by SPSS version 25.

**Results:**

There were one hundred twenty patients. Thirty-eight (31.66%) were females and eighty-two (68.33%) were males. Age range was 13 to 60 years. Ten patients (8.33%) were between 13 – 20 years, sixty five (54.16%) were 21 to 30 years, thirty (25%) were 31 – 40 years, ten (8.33%) were 41 – 50 years and five patients (4.17%) were above 50 years of age. Sixty-two (51.66%) patients had simple appendicitis and fifty-four (45%) had complicated appendicitis like suppurative (26.66%) and (16.66%) gangrenous appendicitis. Negative appendectomy was found in four (3.33%). Hyperbilirubinemia was found (100%) in gangrenous, (94.12%) in suppurative and (12.3%) in catarrhal appendicitis. Enzymes like Alanine transaminase and Aspartate transaminase were raised (50%) in gangrenous, (50%) in suppurative and (9.67%) in catarrhal appendicitis. TLC was raised in 112 (96.55%) out of 116 patients and total leukocyte count was normal in negative appendectomies.

**Conclusions:**

It is concluded that hyperbilirubinemia is strong diagnostic predictor for complicated appendicitis.

## INTRODUCTION

Diagnosis of acute appendicitis is essential for early appendectomy to prevent the complications of appendicitis. Complications of appendicitis are life threatening like appendicular abscess, gangrenous and perforation of appendix. Mostly appendicitis is diagnosed on clinical examination but can be challenging in females and children.^1^ Total leukocyte count is not always a good diagnostic criteria in acute appendicitis but when combined with clinical examination, it is helpful for appendectomy.^2^ C-reactive protein and bilirubin are raised significantly in acute appendicitis (96.6%).^3^ Sensitivity of TLC is more than (90%) in the diagnosis of acute appendicitis.^4^ Raised bilirubin level strongly supports and depicts appendicular perforation. Leukocyte count more than 13000 is found in gangrenous appendicitis.^5^ C-reactive protein to lymphocyte ratio has a diagnostic role in differentiating acute and perforated appendicitis.^6^ Bacterial invasion in appendix leads to transmigration of bacteria and release of pro inflammatory cytokines such as interferon alpha. These reach via superior mesenteric vein to liver and produces inflammation, abscess or dysfunction of liver by changing blood flow to liver. It has been observed that high bilirubin level in acute appendicitis showed predictor of gangrenous or perforated appendix.^7^ Bilirubin is raised badly in perforated appendix.^8^ Hyperbilirubinemia is new investigation for diagnosis of perforated appendix it may be due to hepatocellular cholestasis and hemolytic disease when bacteria overwhelmed to Kupffer cell function. It may cause dysfunction of hepatocytes so markedly that bilirubin is raised but liver enzymes may be raised as well. It also causes decreased excretory function of liver.

Rational of this study was that hyperbilirubinemia in literature is observed to have significant role in diagnosis of complicated acute appendicitis like suppurative or gangrenous or perforated appendix. This study was conducted to find its importance especially in suppurative and gangrenous appendicitis. The objective of the study was to determine role of hyperbilirubinemia in the diagnosis of different stages of acute appendicitis.

## METHODS

Total 120 patients diagnosed as acute appendicitis both males and females above 12 years of age were included. Study was conducted at ward-3, Jinnah Post Graduate Medical Center (JPMC) from June 2019 to June 2020 for one year. Patient’s history and clinical examination were performed in emergency, patient’s complete blood count (CBC), serum liver function test, serum urea, creatinine, sugar were done. X-ray chest and ECG in above 40 years were also carried out. Emergency appendectomy was done. Operative findings like catarrhal, suppurative and gangrenous appendix were noted on performa. Appendix specimen were sent for histopathology examination and findings of histopathology like normal appendix, catarrhal, suppurative and gangrenous appendix were confirmed and noted on performa. Bilirubin above 1.1mg/dl, ALT and AST above 50 IU and TLC above 11000 were considered as high. Hepatitis C and HBsAg were carried out. Patients of known co-morbids like hepatitis, cirrhotic liver disease, gall bladder disease, chronic liver disease, on steroids or analgesics were excluded from the study. Hyperbilirubinemia was related to catarrhal, suppurative and gangrenous appendicitis and association of hyperbilirubinemia was recorded and calculated by SPSS version 25 in frequency and percentages. This study was approved by the Institutional Review Board of JPMC with the reference no. F.2-81/2020-GENL/42867/JPMC.

## RESULTS

There were 120 patients of acute appendicitis. Thirty-eight patients (31.66%) were females and eighty-two (68.33%) were males. Age range was 13 to 60 years. Ten patients (8.33%) were between 13 – 20 years of age, sixty five patients (54.16%) were between 21 to 30 years of age, thirty patients (25%) were between 31 – 40 years of age, ten patients (8.33%) were between 41 – 50 years of age and five (4.17%) were above 50 years of age. Sixty-two (51.66%) patients proved simple as acute catarrhal appendicitis and fifty four (45%)found out to be complicated appendicitis like suppurative (28.33%) and (16.66%) gangrenous appendicitis. Negative appendectomy was found in four patients (3.33%). Patients presented after 24 hours had complicated appendicitis like suppurative and gangrenous appendicitis. Hyperbilirubinemia was found (100%) in gangrenous, (94.12%) in suppurative and (12.3%) in catarrhal appendicitis. Enzyme like Alanine transaminase and Aspartate transaminase were raised in (50%) in gangrenous, (47.05%) in suppurative and (9.67%) in catarrhal appendicitis. TLC was raised in 112 (96.55%) out of 116 patients of acute appendicitis and TLC was normal in negative appendectomy.

## DISCUSSION

Every investigation that can help in the diagnosis of acute appendicitis is valuable. Research has supported that hyperbilirubinemia is more specific marker for both simple and perforated appendicitis than TLC. Although this investigation is not commonly used. Hyperbilirubinemia is specific if appendix is perforated and excludes the other causes of pain in right iliac fossa.^9^

Previously Alvarado score and modified Alvarado score were diagnostic but could not differentiate between different stages of appendicitis. Recently elevation in serum bilirubin was reported in acute appendicitis. Bacterial invasion to appendix and release of tumor necrosis factor alpha, interleukin 6, and cytokines these reach to liver via superior mesenteric vein and raise bilirubin.^10^ In this study bilirubin was raised mostly in complicated appendix like in gangrenous appendix it was 100% and in suppurative it was 94.12%.

**Table-I:** Showed deranged liver functions test in different stages of acute appendicitis.

*Type of Acute Appendicitis*	*No. of patients*	*Hyperbilirubinemia*	*Raised ALT*	*Raised AST*
Acute Catarrhal	62/120	8/62	6/62	6/62
Appendicitis	51.66%	12.90%	9.67%	9.67%
	(42.74 - 60.51)	(6.17 - 23.03)	(4.01 - 19.04)	(4.01 - 19.04)
Acute	34/120	32/34	16/34	16/34
Suppurative	28.33%	94.12%	47.05%	47.05%
Appendicitis	(20.82 - 36.88)	(81.90 - 99.00)	(30.87 - 63.73)	(30.87 - 63.73)
Acute	20/120	20/20	10/20	10/20
Gangrenous	16.67%	100%	50%	50%
Appendicitis	(10.78 - 24.40)	(86.09 - 100.00)	28.86 - 71.14	28.86 - 71.14
Normal	4/120	0/4	0/4	0/4
Appendicitis	3.33%	0.00%	0.00%	0.00%
	(1.06 - 7.80)	(0.00 - 52.71)	0.00 - 52.71	0.00 - 52.71
Total No. of patients		60/120	32/120	32/120
	120	50.00%	26.67%	26.67%
		(41.11 - 58.89)	(19.34 - 35.11)	(19.34 - 35.11)

Figures in parentheses are 95% Confidence Interval

Raised serum bilirubin level is diagnostic marker in appendicular perforation and helpful investigation.^11^ Neutrophil are more than 75% raised it showed high index of suspicion of acute appendicitis in children^12^ to prevent delay in appendectomy. Hyperbilirubinemia was found 100% in gangrenous appendicitis and helpful in immediate decision for appendectomy and it prevents the laparotomy. Because delay in operative treatment can lead to generalized peritonitis and increased morbidity and mortality. Raised lymphocyte count can be diagnostic of ruptured appendicitis if more than 14.8%.^13^ In combination hyperbilirubinemia is a strong predictor of gangrenous appendicitis same findings were noted in this study.

In pregnancy TLC are already high.^14^ In pregnancy ultrasonography of abdomen and hyperbilirubinemia proved diagnostic and decision-making investigation for appendectomy adjuvant to the clinical examination.

Negative appendectomy has lot of complications like wound infection and delayed hospital stay.^15^ In such situation hyperbilirubinemia may help in decision of appendectomy and can prevent the negative appendectomy as well. Negative appendectomy was 3.33% in this study. This was low as compared to literature, it may be due to accurate diagnosis of acute appendicitis and exclusion of children below 12 years of age in this study.

Alvarado score is 99% sensitive.^16^ TLC is raised in 90%,^17^ whereas hyperbilirubinemia was found raised 94.12% in suppurative appendicitis and 100% in gangrenous appendicitis in this study it proved strong predictor of complicated appendicitis as compared to simple appendicitis. In catarrhal appendicitis only 12.90% patient had hyperbilirubinemia in study. Hyperbilirubinemia observed strong predictor in complicated appendicitis in literature and same findings were found in this study.

Another study showed if TLC, C-reactive protein and hyperbilirubinemia were found raised then it proved strong prediction of perforated appendicitis or gangrenous appendicitis.^18^ In this study TLC was raised and hyperbilirubinemia was found 100% in gangrenous appendicitis as well. Antibiotic affects the TLC if already taken.^19^ But bilirubin is helpful in decision making in gangrenous appendicitis.

Other diagnostic investigations for acute appendicitis like ultrasonography, Computerized tomography, Magnetic Resonance Imaging are available in tertiary center, these diagnostic investigations are not available in every primary and secondary care center. These investigations are also expansive as well, these investigations are also time consuming and delay appendectomy. Ultrasound is operator dependent and sometimes Sonologist is not available in primary and secondary center therefore it’s not a reliable tool. These investigations also have limitations because these do not always have accurate results. CT scan abdomen has radiations and gives radiations equal to 500 X-ray chest, which have a risk of cancer of abdominal organs. CT scan is contraindicated in children and pregnancy. Diagnostic role of ultrasonography is still controversial in literature. On other hand TLC, C-reactive protein and serum LFT are available in every primary and secondary health care center. TLC, C-reactive protein and hyperbilirubinemia adjuvant to clinical examination make the easy and early decision for appendectomy. If surgeon is doubtful and has suspicion of appendicitis on clinical examination, then these investigations are helpful for decision making for appendectomy.

Hyperbilirubinemia reduces the risk of negative appendectomy as well. Migratory Pain in right iliac fossa, tenderness and rebound tenderness are strong diagnostic signs for acute appendicitis but if migratory pain is not present and surgeon is suspicious of appendicitis then hyperbilirubinemia has proven to be a strong evidence for acute appendicitis and helps to make decision for early appendectomy. Another study also showed hyperbilirubinemia present in perforated appendix.^20^ In our study same finding was found. In acute appendicitis, there is excretory problem of bilirubin, therefore enzymes are also affected. In this study liver enzymes are also raised but not in all patients. Mostly jaundice is cholestatic type, so hyperbilirubinemia was present predominately in complicated appendicitis. Increase in enzyme also depend on site and severity of hepatocytic injury as well. In acute appendicitis, males are predominately involved more common in between 20 – 30 years of age. The same finding was reported in this study.

### Limitations of the study

Children below 12 years of age were excluded. That could affect results slightly and study was of one-year long duration. We may increase the sample size for further studies.

## CONCLUSION

It was concluded that hyperbilirubinemia is a strong predictor and diagnostic tool for complicated appendicitis. However, it did not show a significant impact in uncomplicated appendicitis.

### Authors’ Contribution:

**KMB:** Data collection, study design, result interpretation, discussion, references and script writing. She is responsible for integrity of research. **SP:** Discussion and result interpretation. **MI:** Study design, result interpretation and drafting. **AM:** Data collection.

## References

[1] Charity C, Shawn G, Rangel J (2016). Overview and diagnosis of acute appendicitis in children. Sem Pediat Surg.

[2] Kamran H, Naveed D, Nazir A, Hameed M, Ahmed M, Khan U (2008). Role of total Leukocyte Count in Diagnosis of acute Appendicitis. J Ayub Med Coll Abbottabad.

[3] Alam B, Malik Z, Ibrar M, Abdullah MT, Waqar S, Zahid M (2014). High total Leukocyte Count in the Diagnosis of Acute Appendicitis. J Med Sci.

[4] Mir MA, Haq I, Manzoor F (2016). Diagnostic Value of Total Leucocyte Count (TLC). C-reactive Protein (CRP) and Bilirubin in Patients with Suspected Acute Appendicitis. Int J Contemp Med Res.

[5] Estrada JJ, Petrosyan M, Barnhart J, Tao M, Sohn H, Towfigh S (2007). Hyperbilirubinemia in appendicitis:a new predictor of perforation. J Gastrointest Surg.

[6] Koyuncu S, Ismail O (2020). The role of C-reactive protein to lymphocyte ratio in the differentiation of acute and perforated appendicitis. Turk J Trauma Emerg Surg.

[7] Khan S (2008). Elevated serum bilirubin in acute appendicitis:a new diagnostic tool. Kathmandu Univ Med J.

[8] Emmanuel A, Murchan P, Wilson I, Balfe P (2011). The value of hyperbilirubinemia in diagnosis of acute appendicitis. Ann R Coll Surg Engl.

[9] Souza ND, Karim D, Sunthareswaran R (2013). Bilirubin;A diagnostic marker for appendicitis. Int J Surg.

[10] Raj RS (2018). Evaluation of Hyperbilirubinemia in Acute Appendicitis. Int J Contemp Med Res.

[11] Goudar BV, Kanchan VR (2020). Elevated serum bilirubin in acute appendicular perforation as a newer serum marker:a diagnostic validation test. Int Surg J.

[12] Rassi R (2019). Acute appendicitis in children under 4 years. Diagnostic Dilemma. Rev Fac Cien Med Uni Nac Cordoba.

[13] Virmani S, Prabhu PS, Sundeep PT, Kumar V (2018). Role of Laboratory Markers in Predicting Severity of Acute Appendicitis. Afr J Paediatr Surg.

[14] Neto AHF, Amorim MMR, Nóbrega BMSV (2015). Acute appendicitis in pregnancy. Rev Assoc Med Bras.

[15] Kabir SA, Sun R, Jafferbhoy S, Karim A (2017). How to diagnose an acutely inflamed appendix;A systematic review latest evidence. Int J Surg.

[16] Macco S, Vrouenraets BC, de Castro SM (2016). Evaluation of scoring systems in predicting acute appendicitis in children. Surgery.

[17] Frountzas M, Stergios K, Kopsini D, Schizas D, Kontzoglou K, Toutouzas K (2018). Alvarado or RIPASA score for diagnosis of acute appendicitis?A meta-analysis of randomized trials. Int J Surg.

[18] Iqbal MN, Ahmad S, Saeed A, Shah MI, Dogar MZI, Mustafa G (2019). Predictive value of total leucocyte count (TLC), bilirubin and C-reactive protein in the diagnosis of gangrenous and perforated appendicitis. Prof Med J.

[19] Ainippully AM, Narayanan SK, Vysakh CN, Arun Preeth V, Somnath P (2017). Role of total leukocyte count in diagnosis of pediatric acute appendicitis:effect of antibiotic administration and duration of onset of symptoms. Int Surg J.

[20] Kanlioz M, Karatas T (2019). The Relationship of Perforated Appendicitis with Total and Direct Bilirubin:Cureus.

